# Report of a novel missense *TDP1* variant in a Pakistani family affected with an extremely rare disorder congenital spinocerebellar ataxia with axonal neuropathy type 1 (SCAN1)

**DOI:** 10.1007/s11033-024-10085-8

**Published:** 2024-11-22

**Authors:** Riaz Ahmad, Filza Sayyad, Muhammad Naeem, Henry Houlden

**Affiliations:** 1 Medical Genetics Research Laboratory, Department of Biotechnology, Quaid-I-Azam University, Islamabad, 45320 Pakistan; 2 Department of Neuromuscular Disorders, UCL Queen Square Institute of Neurology, Queen Square House, London, WC1N 3BG UK

**Keywords:** TDP1, Spinocerebellar ataxia, Spinocerebellar ataxia with axonal neuropathy type 1

## Abstract

**Background:**

Spinocerebellar ataxia with axonal neuropathy type 1 (OMIM: 607250) is an extremely rare autosomal recessive disorder caused by a mutation in the tyrosyl-DNA phosphodiesterase 1 (*TDP1)* gene. Only a single missense variant (p.His493Arg) in this gene has been reported. This variant was found in three Arab families with a possible common founder effect.

**Methods and Results:**

We report a female patient born to a consanguineous Pakistani family segregating autosomal recessive spinocerebellar ataxia with axonal neuropathy type 1. The patient presents additional clinical features distinct from previously reported Arab families including congenital onset of the disease. We performed whole exome sequencing with the patient’s DNA and identified a novel missense variant (NC_000014.9:g.89991982C > T; p.His478Tyr) in exon 13 of the *TDP1* gene. Sanger sequencing was performed to verify the autosomal recessive segregation of the p.His478Tyr variant in the family.

**Conclusion:**

The current study expands both the clinical and mutation spectrum of the *TDP1* associated spinocerebellar ataxia with axonal neuropathy type 1 and increases the body of evidence that supports the pathogenic role of *TDP1* in cerebellar ataxias with peripheral neuropathy.

## Introduction

Defects in the response to DNA double-strand or single-strand breaks underpin different human diseases associated with the nervous system. Throughout nervous system development, internal or endogenous DNA damage mostly triggers apoptosis, although cell replacement can happen from germinal zones inside this fast-proliferating tissue. A faulty mechanism for the detection of DNA damage might allow a cell with a defective genome to become part of the nervous system leading to subsequent cell death and neurodegeneration [1, 2]. Spinocerebellar ataxia with axonal neuropathy type 1 (SCAN1) was first reported in a Saudia Arabian family characterized by gait disturbance, distal amyotrophy, mild dysarthria, moderate ataxia, pes cavus, peripheral axonal sensorimotor neuropathy, hypercholesterolemia and no cognitive impairment. The *TDP1* encoding tyrosyl-DNA phosphodiesterase 1 (TDP1) is the causative gene for SCAN1 and is located on chromosome 14q31-32 [3]. TDP1 actively participates in the repair of DNA strand breaks linked with different DNA termini, the most well-known of them are topoisomerase-1 linked 3’-termini originating from abortive top1–DNA complexes [4].

Topoisomerase-1 or TOP1 alleviates torsional stress in DNA through the generation of a transient intermediate also known as the cleavage complex, in which topoisomerase-1 produces a covalent phosphodiester bond between the 3’-end of a DNA nick and an active site tyrosyl residue [5]. TOP1 combines with DNA in proximity to nicks and altered DNA bases and typically the topoisomerase-1-DNA complexes are transient. However, accumulation of the complexes may cause stalling and loss of ability to repair DNA [6]. TDP1 replaces the stalled fragment of TOP1, generates a TDP1-DNA covalent bond and subsequently hydrolyzes this bond, facilitating the removal of TOP1 peptide from the 3’ terminus and allowing repair of the strand breaks of DNA [7]. If not addressed, TOP1-DNA complexes might be converted into TOP1-associated DNA double-strand or single-strand breaks via collision with the DNA replication or transcription machinery [8]. In this context, TDP1 works as a fail-safe to shield cells from harmful effects or genotoxicity linked with topoisomerase-1-dependent lesions. TDP1 is also able to hydrolyze other DNA single-strand breaks (SSBs), for example, 3’ phosphoglycolate that results from genotoxic stress [9].

Therefore, TDP1 is an important enzyme that functions in DNA repair. The DNA strand breaks are deleterious if not repaired and even develop spontaneous mutations, or might affect gene transcription. These mutations can facilitate cancers and deformation in the peripheral nerves and brain. Homozygous missense mutation such as p.His493Arg in *TDP1* can cause SCAN1 disorder, which is a type of recessive spinocerebellar neurodegenerative disorder [3, 10]. In the current study, we present a novel missense *TDP1* variant identified in a patient born to a consanguineous Pakistani family affected with autosomal recessive SCAN1.

## Material and Methods

### Ethical approval and human participants

The research conducted on rare case of spinocerebellar ataxia with axonal neuropathy 1 was approved by the ethical board of Quaid-I-Azam University, Islamabad (QAU/IRB/505) and the clinical history and consent form were obtained from the family who are from Azad Kashmir, Pakistan. DNA from the patient, sibling and parents were extracted using standard protocol.

### Genetic Evaluation

#### Whole Exome Sequencing (WES)

For identification of a causative variant, WES was performed through Agilent SureSelect Human All Exome V6 Kit (Agilent Technologies, Santa Clara, CA, USA). The sequencing reads were obtained against the reference genome of humans that was aligned by Burrows-Wheeler Aligner v0.7.17 (http://bio-bwa.sourceforge.net/bwa.shtml). The obtained files of BAM were then sorted through SAMtools v1.8 (https://github.com/samtools/samtools/releases/tag/1.8) and duplicated reads were marked by Picard (v2.18.9) (http://sourceforge.net/projects/picard/). Genome Analysis Toolkit (GATK) v4.0 (https://gatk.broadinstitute.org/) was done for the genotyping. Annotate Variation or ANNOVAR (https://annovar.openbioinformatics.org/en/latest/) was carried out while FILTUS (http://folk.uio.no/magnusv/filtus.html) was used for variant filtration. Furthermore, after annotation, the created file was obtained in CSV format and was filtered to explore the disease-causing pathogenic variants. Our targeted region was the coding (exons) and splice regions (both donor and acceptor sites). Based on phenotypic characteristics and genealogical analysis, our focus lies on rare variants with minor allelic frequency < 0.01% as cataloged on public databases such as 1000 Genomes project (https://www.internationalgenome.org/), Exome Aggregation Consortium (https://gnomad.broadinstitute.org/), NHLBI Exome Variant Server (http://evs.gs.washington.edu/EVS/) and Complete Genomics 69 (https://www.completegenomics.com/). The inheritance pattern encompassing heterozygous, homozygous and compound heterozygous were also taken into consideration during filtration. In addition, probable and pathogenic variants were given precedence following the American College of Medical Genetics and Genomics (ACMG) guideline.

#### Homozygosity mapping and Sanger sequencing

**Fig. 1:**
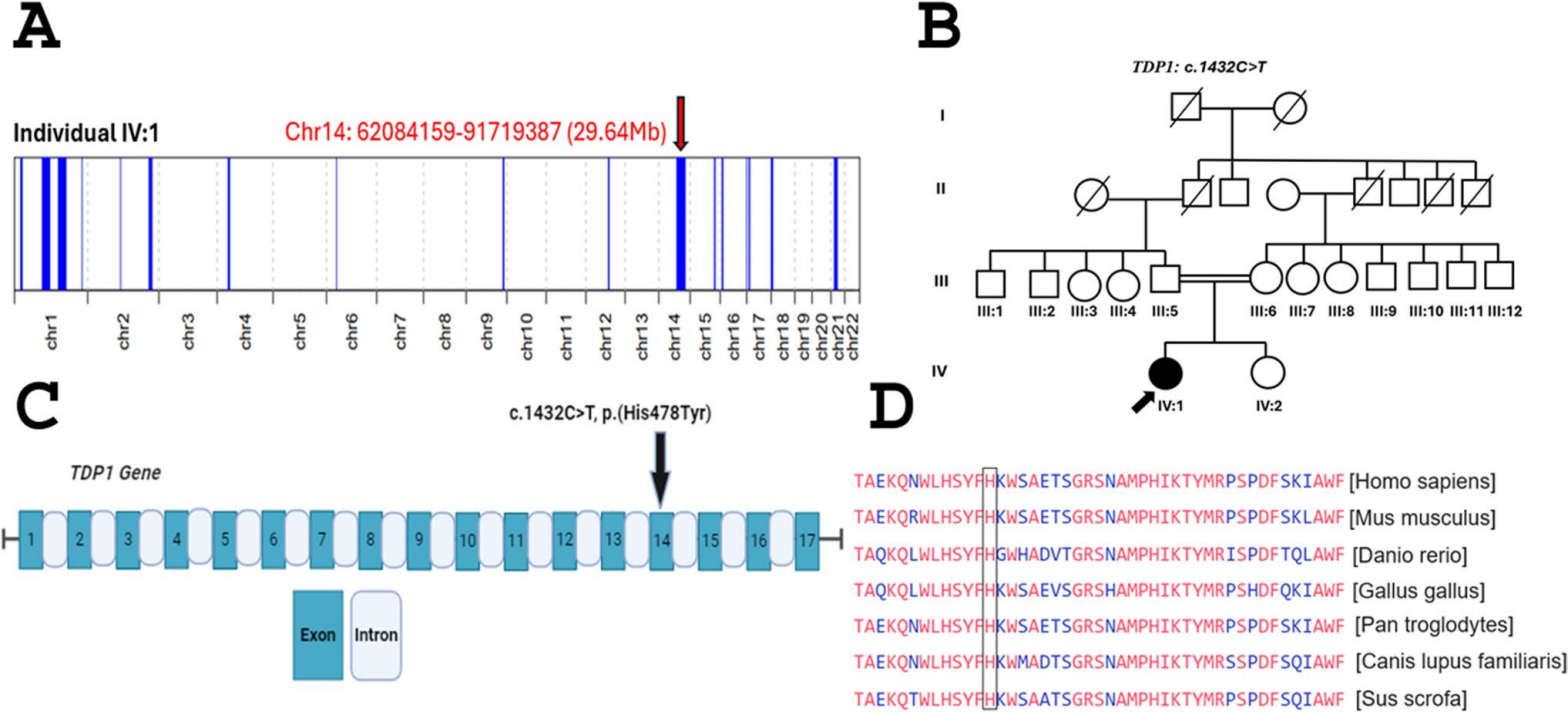
**A** Homozygosity mapping through AutoMap shows a large homozygous region in the proband on chromosome 14 harbouring *TDP1*. **B** Pedigree of the proband affected with SCAN1. **C** Exonic location of the identified variant. **D** Conservation of the amino acid His478 in TDP1 in various species

AutoMap (https://automap.iob.ch/process) was used for the novel variant with default settings (Fig. 1A). AutoMap is a potent tool for generating homozygosity mapping directly using VCF (Variant Call Format) files of WGS or WES [11].

A pair of forward (TCCTGTTACCTTCTTGCTGTT) and reverse primer (TTTGTCTTTCTTGGCATGCC) were designed through the Primer3Web (version 4.1.0) tool (https://primer3.ut.ee/) and used to amplify the targeted exon 13 based on WES shortlisting. Through standard protocol, co-segregation analysis was done by Sanger sequencing (ABI-3730 DNA analyzer). The results were visualized with Sequencher 5.0 software.

#### Tools for protein modeling of wild-type and mutant TDP1 protein

AlphaFold Notebook, Chimera-X, UCLA-DOE LAB- SAVES v6.0, Missense 3D, Mupro and Galaxy Refine were used for the protein modeling of TDP1 protein.

## Results

**Fig. 2:**
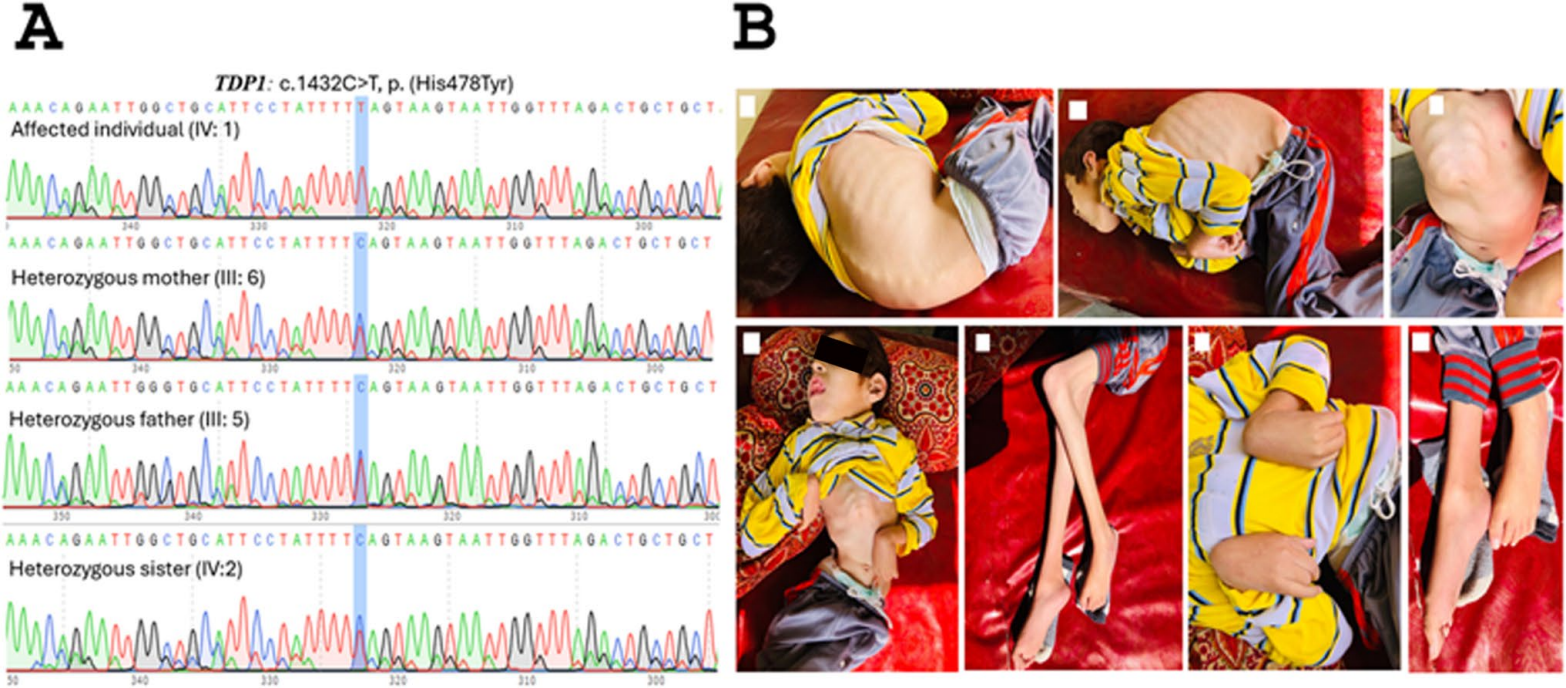
**A** Segregation analysis of the identified variant in the proband’s family through Sanger sequencing. **B** Phenotypic representation of the proband (IV:1)

We enrolled a Pakistani family in this study after IRB approval and consent was signed having neurological features. The proband has consanguineous parents and phenotypic manifestations are shown in Figs. 1B and 2B, respectively. The disease with generalised hypotonia was observed at birth of the affected individual (IV:1). The patient failed to achieve any gross motor development milestones. She also has impaired hearing and vision, which indicates significant sensory deficits while no speech is attributed to sensory and motor impairments. She later developed seizures at 9–10 months of age. Examination reveals severe muscle wasting with kyphoscoliosis and flexion contractures at elbows, wrist and knees and plantar flexion contractures at ankles. EEG examination showed slow theta waves suggestive of generalised cortical dysfunction**.** Overall, the proband is severely affected by impaired physical, sensory and motor development from birth**.** Most of the symptoms are matched with reported cases of spinocerebellar ataxia, autosomal recessive with axonal neuropathy 1 (OMIM 607250).

Through whole exome sequencing, we identified a likely pathogenic variant in exon 13 of the *TDP1* (NC_000014.9: g.89991982C > T; p.His478Tyr) that was found highly conserved among different species (Fig. 1C & 1D). The segregation analysis through Sanger sequencing verified the autosomal recessive inheritance pattern of the disease in the family (Fig. 2A). The variant pathogenicity was supported by different in silico tools like polyphen-2 (possibly damaging (0.721), aggregated prediction (deleterious (0.8), VARITY (deleterious (0.48), MutationTaster (disease-causing (1), GenoCanyon (deleterious (1), and DANN (deleterious (1), dbscSNV (pathogenic supporting (0.9933), EIGEN (pathogenic supporting (0.7201) and VarSome (PP3: pathogenic very strong). Based on the ACMG guideline, our variant is likely pathogenic supported by the following criteria: PP2 (a novel missense variant is considered for pathogenicity), PP3 (computational evidence for pathogenicity), PP5 (recent reports as evidence of pathogenic effect and PM2 (low frequency in public databases if recessive).

**Fig. 3:**
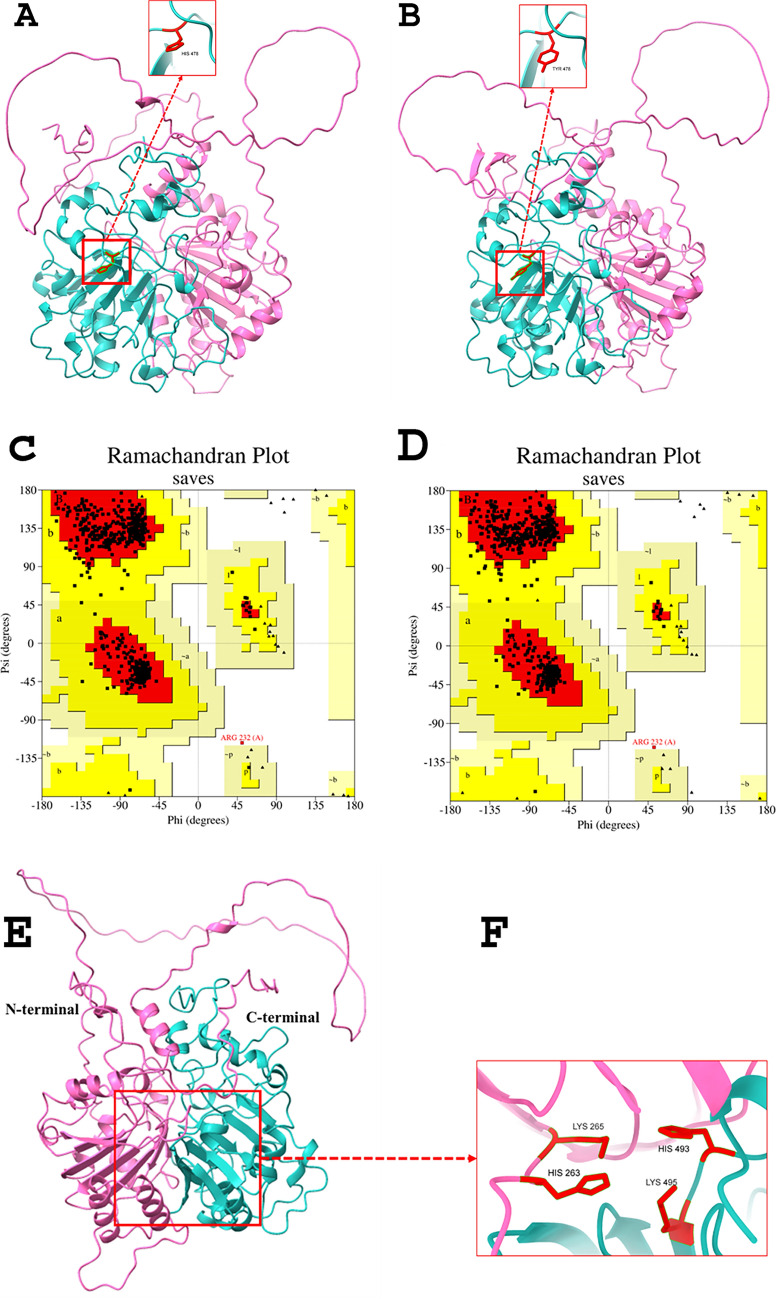
In silico analysis of the TDP1 protein variants. Wild-type **A** and mutant **B** TDP1 protein structures predicted by Alphafold. Panels **C** and **D** depict Ramachandran wild-type plot and mutatnt plot, respectively by UCLA SAVES v6.0. The red area is the most favorable, dark yellow is the additional allowed region, light yellow is the generously allowed region and white is the disallowed region. Glycine is depicted by triangles. **E** The active site of TDP1 protein. The pink and light sea green colors represent the N-terminal and C-terminal, respectively. **F** Active site important residues are shown as sticks in red color. Lys-265 and His-263 are in N-terminal and His-493 and Lys-495 are present in the C-terminal domain of the TDP1 protein

The amino acid sequence of the *TDP1* gene was downloaded from Ensembl (ENST00000335725.9) and submitted to Alphafold Colba Jupiter Notebook (accessed on 1st July 2024). Models were generated by selecting the PDB database as a template, 12 recycles and four seeds. The best models with pLDDT 80.6 and 80.9, pTM 0.805 and 0.81 for wild-type and mutant TDP1, respectively, were selected for structural analysis (Fig. 3A & B). Structures were further refined by Galaxy Refine (https://galaxy.seoklab.org/cgi-bin/submit.cgi?type=REFINE; accessed on 1st July 2024) and validated by Ramachandran plots using SAVES v6.0 (https://saves.mbi.ucla.edu). Ramachandran plot generated by SAVES v6.0 (https://saves.mbi.ucla.edu; accessed on 1st July 2024) showed that 94.7% of the residues are in the most favorable region, 5.2% in the additional allowed region and 0.2% in the disallowed region of the wild-type protein. However, mutant protein has slightly better results: 95.4% of the residues are in the most favorable region, 4.4% in the additional allowed region and 0.2% in the disallowed region. These results suggest a well-folded protein structure that may exhibit some flexibility and disorder (Fig. 3C and 3D).

TDP1 belongs to the phospholipase D superfamily and as a monomer. The N-terminal domain is in the first 350 residues and showsprotein-protein interactions as well as regulatory functions. The C-terminal domain is in residues 350–608. Both domains have conserved HKD motifs that form active sites for topoisomerase catalyzed formation, and subsequent hydrolysis, of the TDP1-DNA complex. His-263 and His-493 are crucial N-terminal and C-terminal residues respectively which show the nucleophilic attack on the topoisomerase and DNA complex releasing the covalent bond [12, 13] as shown in Figs. 3E & 3F.

**Fig. 4:**
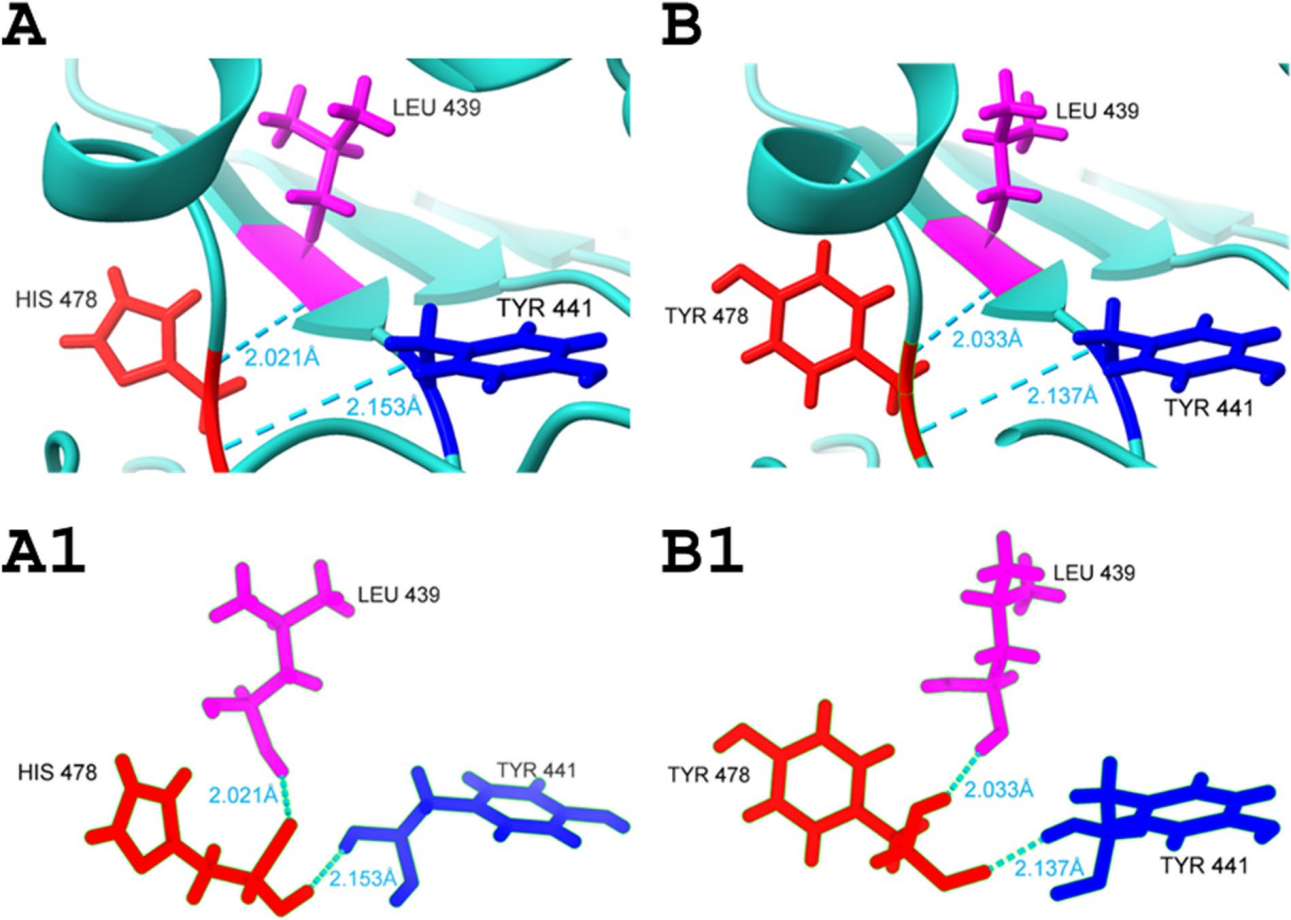
Interactions among wild-type and mutant residues. A and A1: wild-type TDP1 protein with His-478 and its interactions with neighboring amino acids. B and B1: mutant TDP1 protein with Tyr-478 and its interactions with neighboring amino acids. Wild-type and mutant residues are represented by red-colored sticks. Dotted light sea green lines are the hydrogen bonds with distance mentioned in angstrom units

Histidine is a polar, charged residue and has an imidazole ring which acts as both acidic and basic during catalysis. In TDP1 upon substitution at position 478, it gets replaced by tyrosine which is a polar, uncharged amino acid. Visualization in ChimeraX (https://www.cgl.ucsf.edu/chimerax/) describes wild-type and mutant residue (His-478 and Tyr-478) making H-bonds with Leu-439 and Tyr-441 using a cutoff value of 4Å (Fig. 4). Further analysis by Mupro (https://mupro.proteomics.ics.uci.edu; accessed on 3rd July 2024) and Missense 3D (http://missense3d.bc.ic.ac.uk; accessed on 3rd July 2024) showed that at the 478 position, wild-type residue is not exposed so it delineates breakage in the side chain buried H-bond decreasing protein stability (ΔΔG = -1.072) and inducing conformational changes in the TDP1 protein.

## Discussion

The phenotypic features of SCAN1 include a combination of progressive ataxia (without ocular motor apraxia), cerebellar atrophy and co-occurring distal symmetrical sensorimotor axonal neuropathy. There are restricted clinical and genetic reports of *TDP1* gene-related SCAN1 and therefore, the phenotypic spectrum can't be fully ascertained [10]. In the current study, we present clinical and genetic investigation of a SCAN1 patient from a consanguineous Pakistani family. Through whole exome and Sanger sequencing techniques, we identified a novel missense likely pathogenic *TDP1* variant (NC_000014.9: g.89991982C > T; p.His478Tyr) in the biallelic form of segregation. Protein modeling studies of the TDP1 p.His478Tyr mutated protein suggest that disrupted hydrogen bonding and confirmational changes significantly reduce protein stability. Our findings indicate that substitution may impair the enzyme function, potentially contributing to disease-related dysfunction in the DNA repair processes.

According to the literature, only seven individuals affected with SCAN1 from three unrelated arab families have been documented so far. A missense *TDP1* variant p.His493Arg has been reported underlying SCAN1 phenotype in these three families [3, 10]. The clinical features of the seven patients of these three arab families and one patient of our Pakistani family are presented in Table 1. Our patient of Pakistani origin has phenotypic features of SCAN1 previously reported in the Arab families but has few distinct features as well. Most importantly, the Pakistani patient had disease onset from birth and had severe kyphoscoliosis. Moreover, the patient presented with seizures at the age of nine months. The disease onset in the Saudi kindred [3] and two Omani families [10] was in late childhood and adulthood, respectively. The cognitive function in the Saudi family was normal while mild cognitive impairment was reported in the Omani families. In contrast, the Pakistani patient had moderate cognitive impairment.

**Table 1:** Clinical comparison of Saudia Arabian, Omani and Pakistani patients

	Saudia Arabian Family [3] Variant: p.His493Arg			Omani family 1 [10] Variant: p.His493Arg	Omani Family 2 [10] Variant: p.His493Arg			Pakistani Family [current study] Variant: p.His478Tyr
Patients	1	2	3	1	1	2	3	1 (IV:1)
Gender	M	M	F	F	M	M	F	F
Onset (Years)	13y	13y	15y	24y	25y	24y	26y	Congenital
Gait Abnormality	+	+	+	+	+	+	+	+
Hypotonia	+	+	+	+	+	+	+	+ (generalised)
Limbs Incoordination	+	+	+	+	+	+	+	+ (Severe)
Speech deficit	N/A	N/A	N/A	N/A	N/A	N/A	N/A	+
Dysarthria	+	+	+	+	+	+	−	+
Seizures	−	−	−	−	−	−	−	+ (onset at 9 months)
Cognitive Impairment	−	−	−	+	+	+	+	+ (Moderate)
Kyphoscoliosis	−	−	−	−	−	−	−	+ (Severe)
Vibration sense	+	+	+	+	+	+	+	+
Hearing loss	N/A	N/A	N/A	N/A	N/A	N/A	N/A	+
Pes cavus	+	+	+	+	−	−	−	+

Geraud et al. recently generated human SCAN1 cells to understand the molecular bases underlying phenotypic features of SCAN1 disease. They introduced a pathogenic TDP1 variant c.A1478G in SCAN1 cells by CRISPR-Cas9 [3, 10]. The TDP1 p.His493Arg mutated protein showed drastically decreased expression in SCAN1 cells. It was further noted that *TDP1* mRNA level was decreased preferentially as compared to pre-mRNA level indicating that a decrease in *TDP1* transcript stability is responsible for the decreased expression of TDP1 protein instead of decrease in *TDP1* transcription. Moreover, it was found that very low TDP1 activity in SCAN1 cells was due to both low levels of mutated TDP1 protein and reduced catalytic activity. TDP1 deficiency causes increased production of double strand breaks and their defective repair leading to their accumulation in SCAN1 cells. These observations may suggest SCAN1 phenotype results due to loss of function of TDP1. However, it was observed that double strand breaks could be repaired by a TDP2-mediated compensatory pathway in the absence of TDP1 but not in the presence of TDP1 p.His493Arg mutated protein. Therefore, it was suggested that a gain of function rather than loss of TDP1 mutated protein activity hampers a TDP2-dependent backup pathway leading to defective repair of double strand breaks [14]. We recommend similar SCAN1 model studies with the novel *TDP1* p.His478Tyr variant to further investigate molecular mechanisms underlying disease pathogenesis and variable phenotypic severity.

## Conclusion

The clincal heterogeneity observed in our Pakistani patient and underlying novel *TDP1* p.His478Tyr variant expands restricted clinical and mutation spectrum of SCAN1. Our study supports the role of *TDP1* in SCAN1 pathogenesis and recommend inclusion of *TDP1* screening for diagnosis of neurodegenerative atxias. Furthermore, the human cell model should be encouraged for our novel missense variant to further elucidate the disease pathogenesis.

## Data Availability

Not applicable.
